# How to Summarize the Safety Profile of Epoetin Alfa Versus Best Standard of Care in Anemic Patients With Metastatic Breast Cancer Receiving Standard Chemotherapy?

**DOI:** 10.1200/JCO.2016.67.2055

**Published:** 2016-08-08

**Authors:** Takahiro Hasegawa, Hajime Uno, Lee-Jen Wei

**Affiliations:** Takahiro Hasegawa, Shionogi & Co, Ltd, Osaka, Japan; Hajime Uno, Dana-Farber Cancer Institute, Boston, MA; Lee-Jen Wei, Harvard University, Boston, MA

## To the Editor:

Leyland-Jones et al^1^ conducted an open-label, noninferiority study to evaluate the impact of epoetin alfa (EPO) on tumor outcomes when used to treat anemia in patients who received chemotherapy for metastatic breast cancer. The primary end point was progression-free survival (PFS) on the basis of the investigators’ assessments. The study was designed on the basis of the difference between groups as measured by the hazard ratio (HR; EPO *v* best standard care [BSC]), with a noninferiority margin of 1.15. A total of 1,650 PFS events would provide > 80% power with a one-sided type I error of 0.025 to rule out a 15% HR increase. The study was conducted from March 2006 to July 2014, and at the end of study, 1,659 events had been observed. Estimated HR was 1.089, with a 95% CI of 0.988 to 1.200. The observed upper bound exceeded the prespecified noninferiority margin of 1.15. The authors concluded that “Overall, this study did not achieve the noninferiority objective in ruling out a 15% increased risk in PD or death.”^1^

HR is a ratio of two hazard functions over time. Hazard, which is not a probability measure, is commonly misinterpreted as a risk of an event of interest. The observed upper bound, 1.20, of the above 95% CI does not mean that EPO has a 20% risk of increase versus BSC. In fact, it is difficult to interpret the HR in clinically meaningful terms without a hazard function estimate available from BSC. The hazard function, by itself, is difficult to estimate well without a model and difficult to interpret clinically. This issue has been extensively discussed in the clinical and statistical literature, especially for evaluating the safety of a drug or device.^2-4^ The summary measure using HR for this rather lengthy study does not help us to assess the value of EPO under a risk–benefit perspective.

An alternative is to use the restricted mean survival time (RMST) as the summary measure to quantify the group difference.^2-4^ For the present case, survival means PFS. Although the patient-level observations from the study by Leyland-Jones et al^1^ are not publicly available, we used a well-established computer algorithm to scan the Kaplan-Meier (KM) curves presented in their Figure 2A and reconstructed the observed individual times to progression and/or death.^5^ The resulting KM curves and HR estimates with these reconstructed observations are closely matched with the original counterparts reported in the article. With these data, an estimated RMST for PFS ≤ 48 months for EPO is the area under the KM curve in Figure 2A by 48 months, which is 9.9 months. That is, future patients who receive EPO with 48 months of follow-up would achieve a PFS of an average of 9.9 months. For BSC, the RMST estimate is 11.4 months. The difference (BSC − EPO) is 1.5 months (95% CI, 0.5 to 2.6; *P* < .004) in favor of BSC. This difference, coupled with an RMST of 11.4 months for BSC, provides a clinically meaningful interpretation. In any event, when quantifying a group difference with a summary measure, it is informative to have a reference value from the control arm for decision making to assess the benefit and safety profile of a treatment strategy.

There is an ongoing randomized phase III study of darbepoetin versus BSC (NT00858364), for anemia secondary to platinum-based treatment of stage IV non–small-cell lung cancer. We hope that the investigators of the study would consider a sensitivity analysis using the RMST summary measure to further inform the benefit–risk profile of erythropoietin-stimulating agents in the oncology setting.

For future patients’ treatment, we may need more information beyond presenting an overall summary measure for the treatment difference. For such a relatively large study as the present one by Leyland-Jones et al,^1^ it would be important to use information from the patient’s baseline variables to identify a subgroup, if any, of patients who would not have safety concerns, but would benefit from EPO.^6^
